# Evaluation of proximal slicing in primary maxillary incisors with proximal caries- a retrospective cohort study

**DOI:** 10.1186/s12903-023-03648-x

**Published:** 2023-11-21

**Authors:** Avia Fux-Noy, Tamar Goldberg, Aviv Shmueli, Elinor Halperson, Diana Ram, Esti Davidovich, Moti Moskovitz

**Affiliations:** 1 Department of Pediatric Dentistry, Hadassah Medical Center, Faculty of Dental Medicine, Hebrew University of Jerusalem, P.O.B. 12272, Jerusalem, 9112102 Israel; 2 Undergraduate student, Faculty of Dental Medicine, Hebrew University of Jerusalem, Jerusalem, Israel

**Keywords:** Proximal caries, Primary incisors, Minimal intervention, Proximal slicing

## Abstract

**Background:**

Modern management of dental caries should be more conservative and include early detection of lesions and active surveillance, in order to apply preventive measures and carefully monitor for signs of arrest or progression. Proximal slicing was suggested for nonrestorative caries treatment for primary incisors. The aim of the study was to examine the success of proximal slicing in primary maxillary incisors in arresting caries progression.

**Methods:**

A retrospective cohort study. Data were collected from medical records of patients who had undergone proximal slicing in primary maxillary incisors with a follow-up period of at least 6 months. Treatment was considered a success when no further invasive clinical intervention was required within the follow-up period of at least 6 months. Treatment was considered a failure when further invasive clinical intervention was needed during the follow-up period (restoration, crown, or extraction). Additional variables included were the patient’s gender, treated tooth, treated surface, age during their first visit to the clinic, age during slicing treatment, follow-up period, number of follow-up visits, and number of fluoride applications and additional slicing during follow-up.

**Results:**

Seventy-one patients were included in the study. Proximal slicing was successful in 76% of participants with a follow-up of at least 6 months. Success was associated with older age at the first dental visit (3.5yo vs. 2.5yo, p = 0.0011) and age when proximal slicing was performed (4yo vs. 3yo, p < 0.001).

**Conclusion:**

Proximal slicing may successfully arrest proximal caries in primary maxillary incisors.

## Background

Early childhood caries (ECC) in preschool children remains a major problem in both developed and developing countries. Prevention of the progress of the ECC can be achieved with restorations, diet counselling, plaque control and the use of preventive agents like topical fluorides [1, 2]. The management of ECC often requires extensive restorative treatment at an early age [1, 2]. Those treatments require local anesthesia and the use of a rotatory handpiece, which can initiate negative behavior in children [3]. General anesthesia, deep sedation, or moderate sedation may be required at times since young children lack the ability to cope with the extensive treatment procedures [1, 2]. Restorative dentistry has little to no long-term impact on oral bacteria like S. mutans populations; hence, the clinical outcomes for the treatment of ECC are poor. A large portion of treated children experienced new caries lesions within 6–24 months after initial dental treatment [2].

The approach of restoring primary teeth has been questioned [4, 5] since a large portion of primary teeth with untreated carious lesions exfoliates without showing any symptoms [6]. Minimum intervention dentistry (MID) is a reaction to the ineffectiveness of the traditional surgically driven approach to managing enamel and dentin caries. Using the core principles of MID allows a comprehensive patient/family assessment, with early diagnosis of carious lesions, a reliable caries risk assessment, implementation of effective preventive measures, and minimally harmful restorative care. Nowadays, the focus for the management of active lesions is changing the plaque biofilm from a cariogenic state into a non-cariogenic state. The plaque biofilm is left open to the oral environment but is managed in such a way that it changes to a healthy state [7].

**Fig. 1:**
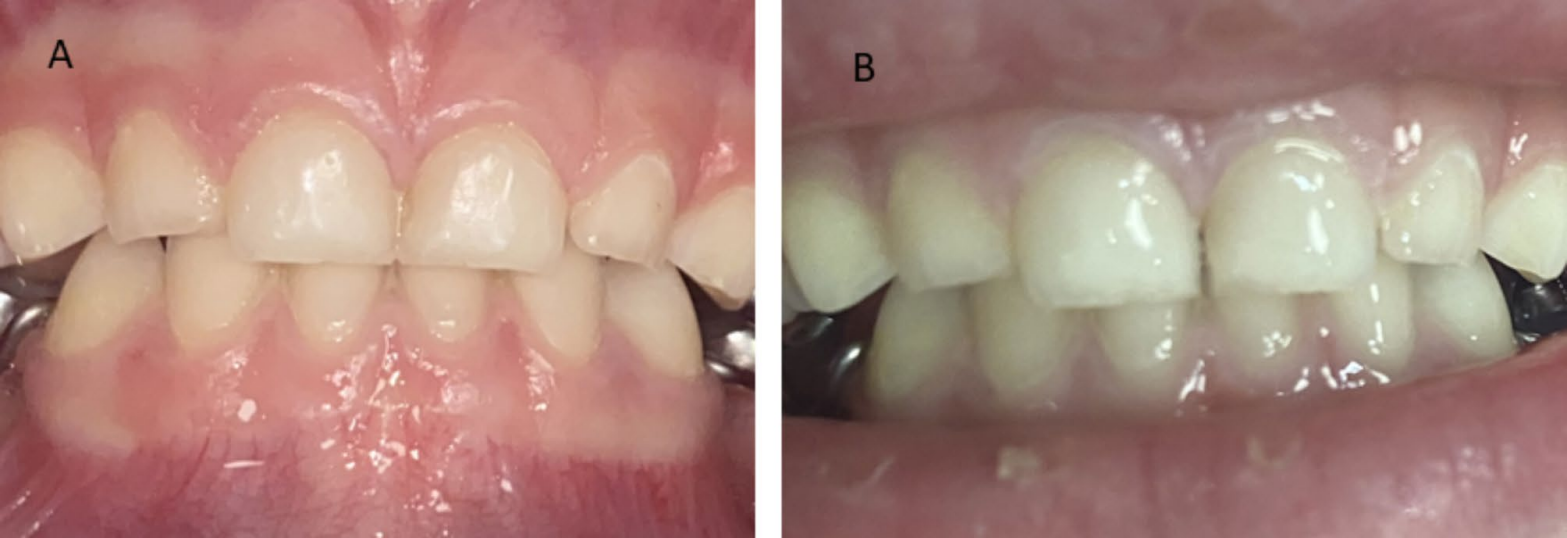
Clinical appearance of maxillary primary central incisors before slicing (**A**) and after slicing (**B**)

Nonrestorative dentin caries treatment was suggested for primary molars [4, 5, 8, 9]. In this technique, no caries is removed but the cavity is opened to allow the lesion to be brushed by parent and child, thus altering the biofilm through continual disruption/cleaning. In anterior teeth, proximal slicing was suggested by Peretz and Gluck [10]. They reported that the progression of ECC was arrested in the vast majority of patients after the preventive regimen, which included hygiene and proper feeding instructions, mesial slicing, and supervised professional topical fluoride treatment. It is the only study describing proximal slicing in anterior teeth. The method includes proximal surface grinding with a high-speed bur to remove the contact point in enamel, making the cavity accessible for plaque removal (Fig. 1). Carious dentine is not removed from the pulpal wall. This procedure is performed without local anesthesia.

The purpose of the present study was to examine the success of proximal slicing in primary maxillary incisors in arresting enamel and dentin caries progression.

## Methods

This is a retrospective cohort study based on data from computerized medical records. The study group was comprised of patients from the Department of Pediatric Dentistry, Hadassah University Medical Center, Jerusalem, who were treated with proximal slicing of maxillary primary incisors with proximal caries, enamel only or enamel and dentin not exciding the outer third of dentin in the radiograph. Inclusion criteria also included a follow-up period of at least 6 months. Exclusion criteria included dental trauma prior to or during the follow-up period. Clinical diagnosis and treatment were performed by three pediatric dentistry specialist instructors in the residency program. The following data were extracted from the medical records:

- Patient’s age at the first visit to the dental clinic.
- Patient’s age when treated with proximal slicing.
- Patient’s gender.
- The tooth on which the treatment was performed, or in cases when the procedure was performed on several teeth, one tooth per patient was randomly included in the study.
- The surface of the tooth where the treatment was performed (mesial/distal).
- Duration of the follow-up period (in months).
- Number of dental follow-up visits during the follow-up period.
- Number of fluoride applications during the follow-up period.
- Number of re-slicings during the follow-up period.
- Evaluation of the treatment as success or failure. Treatment was considered a success when no further invasive clinical intervention, such as restoration, strip crown, or extraction, was needed within the follow-up period of at least 6 months. Treatment was considered a failure when further invasive clinical intervention, such as restoration, strip crown, or extraction, was needed during the follow-up period.

### Ethics

The study was approved by the Institutional Human Subjects Ethics Committee (HMO-0288-19) and conforms to the declaration of Helsinki. Institutional Human Subjects Ethics Committee of Hadassah Medical Organization IRB, Jerusalem waived the need for informed consent due to a retrospective cohort study.

### Statistical analysis

Data analysis was performed using SAS software, version 9.04 (SAS Institute Inc, Cary, North Carolina). The categorical and dichotomous variables were presented using frequencies and percentages. The continuous variables were described by the mean and standard deviation (SD). A comparison of the continuous independent variables among patients in whom the method was successful as compared to those in whom the method failed was performed using a T-test for independent samples. Examining the association between the categorical independent variables and the success of the method was carried out using the Chi-Square test. Significance was defined as p < 0.05.

## Results

Medical records included 156 patients in whom a procedure of proximal slicing was performed, of whom 81 did not meet the inclusion criteria as slicing was performed in teeth other than maxillary incisors or the follow-up period was shorter than 6 months. Of the remaining 75 patients, four were excluded due to dental trauma during the follow-up period. The final study group included 71 primary maxillary incisors with proximal enamel and dentin caries, treated with proximal slicing in 71 children aged 1–6 years (mean 3.30 years, SD 1.11). The method was successful in 76.1% of the patients. Patient and treatment characteristics are provided in Table 1.

**Table 1:** Patient and Treatment Characteristics

Variable		n (%)	Range	Mean (SD)
Patient’s age at first visit (years)			1.3–5.5	3.30 (1.11)
Patient’s age when treated (years)			1.9–5.8	3.77 (1.05)
Gender	Male	35 (49.3)		
	Female	36 (50.7)		
The incisor tooth	Right central	33 (46.5)		
	Left central	30 (42.3)		
	Right lateral	4 (5.6)		
	Left lateral	4 (5.6)		
Tooth surface	Mesial	68 (95.8)		
	Distal	3 (4.2)		
Follow-up period (months)			6–63	24.89 (13.64)
Dental examinations visit during the follow-up period			1–6	2.83 (1.30)
Fluoride application during follow-up			0–5	1.32 (1.20)
Re-slicing during follow-up			0–2	0.15 (0.40)
Method success	Yes	54 (76.1)		
	No	17 (23.9)		
Failure type (n = 17)	Restoration	16 (94.2)		
	Extraction	1 (5.8)		

Variables associated with method success were age at the first dental visit (p = 0.0011), age at the slicing procedure (p < 0.001), and duration of the follow-up period (p < 0.001) (Table 2). No association was found between gender, the treated tooth, the treated surface, the number of dental follow-up visits, the number of fluoride applications and re-slicing, or the success of the method (Table 2).

**Table 2:** Variables associated with slicing success

	N	Success		Odds Ratio	P value
		No	Yes		
Patient’s age at first visit (years), Mean (SD)	71	2.56 (0.81)	3.54 (1.09)		0.0011
Patient’s age when treated (years), Mean (SD)	71	3.05 (0.74)	4.00 (1.03)		< 0.001
Gender					0.1450
Male, n(%)	35(49.3)	11(31.4)	24(68.6)	Reference	
Female, n(%)	36(50.7)	6(16.7)	30(83.3)	2.3(0.7,7.1)	
Incisor tooth treated					0.3092
Right central, n(%)	33(46.5)	6(18.2)	27(81.8)	Reference	
Left central, n(%)	30(42.3)	7(23.3)	23(76.7)	0.7(0.2,2.5)	
Right lateral, n(%)	4(5.6)	2(50.0)	2(50.0)	0.2(0.0,1.9)	
Left lateral, n(%)	4(5.6)	2(50.0)	2(50.0)	0.2(0.0,1.9)	
Tooth surface treated					0.3207
Mesial, n(%)	68(95.8)	17(25.0)	51(75.0)		
Distal, n(%)	3(4.2)	0(0.0)	3(100.0)		
Follow-up period (months), Mean (SD)	71	14.24 (7.54)	28.24 (13.44)		< 0.001
Dental examinations visit during the follow-up period, Mean (SD)	71	2.41 (0.62)	2.96 (1.43)		0.1277
Fluoride application during follow-up, Mean (SD)	71	1.12 (0.78)	1.39 (1.31)		0.4220
Re-slicing during follow-up Mean (SD)	71	0.00 (0.00)	0.20 (0.45)		0.0679

## Discussion

The study found that performing proximal slicing in primary maxillary incisors resulted in a success rate of 76% after at least 6 months of follow-up. Treatment was considered a success when no further invasive clinical intervention was needed, meaning control of lesion activity and arrest of caries progression. Proximal slicing should be accompanied by a preventive regimen, which includes hygiene and proper feeding instructions, and supervised professional topical fluoride treatment [10]. Fluoride is a key factor in oral health promotion and caries prevention. Fluoride treatment aims to increase fluoride incorporation into enamel by prolonging fluoride-tooth surface contact. Fluoride varnish treatment effectively arrests caries by inhibiting demineralization, resulting in highly significant caries reductions [11]. In the current study, the success of slicing was not associated with fluoride application, probably due to the small number of children in whom fluoride application was performed during the follow-up period. However, due to the retrospective nature of the study, fluoride exposure at home and tooth brushing habits were not examined. In addition, there was no difference in the number of follow-up visits between patients for whom the method was successful and those for whom it failed. Follow-up visits are scheduled according to the patient’s caries risk assessment, based on a child’s age, social/behavioral/medical considerations, protective factors, and clinical findings [12].

Enamel and dentine caries lesions are twice as prevalent on the mesial surface of maxillary primary incisors as on distal surface [13]. No difference was found between the surfaces affected by caries since most of the affected surfaces in the current study were mesial. This method may also be effective for the distal surfaces of the maxillary primary central incisors but further studies are required. It is important that future studies will also investigate different tooth types such as maxillary primary lateral incisors and mandibular primary incisors.

Success was found to be related to the age of the patients. The American Academy of Pediatric Dentistry recommends that the first visit to the dentist be at the time of the eruption of the first tooth and no later than 12 months of age [14]. The early dental visit to establish a dental home provides a foundation upon which a lifetime of preventive education and oral health care can be built. Considering these recommendations, it was surprising that among the children in whom the method failed, the age of the first visit was younger than in those in whom the method was successful. It is possible that a combination of insufficient oral hygiene with cariogenic feeding from a bottle or breastfeeding caused younger children to be at higher risk of caries and therefore the success of the method was lower for them [12]. However, no data were collected regarding oral hygiene and nutrition.

The mean follow-up period in the group of children in whom the treatment was defined as a success was 28 months as compared to 14 months in cases where the treatment failed. It can be concluded that most of the failures were detected in the first year after slicing. The majority of failures were considered minor since the tooth was restored and function and esthetic retained. Only one tooth had to be extracted due to caries progression and pain. In addition, the restorative treatment was carried out as the child was older and could tolerate the dental treatment better.

Re-slicing was performed during the follow-up only for a small number of participants for whom the mesial gap was closed during the follow-up period. The need for re-slicing was not considered a failure since it is considered a minimal invasive treatment.

### Study limitations

First, it lacks a control group of children with proximal caries in the primary maxillary incisors that were actively monitored without performing proximal slicing. Second, due to the retrospective nature of the study, it was not possible to examine the influence of the patients’ hygiene and diet habits on the results. A third limitation is the lack of calibration between the clinicians who made the clinical decisions about lesion diagnosis and the criteria they used for making their diagnostic and treatment decisions. In addition, mainly mesial surface of the central primary maxillary incisor was examined.

## Conclusions

Based on this retrospective study, it appears that proximal slicing, of the contacting mesial surfaces of maxillary primary central incisors, with enamel and dentinal caries lesions, may positively aid the arrest of progression of these lesions. Proximal slicing as a minimally invasive treatment for proximal caries in primary maxillary incisors may be a successful method for caries lesion arrest, preventing or delaying invasive treatment procedures. Most treatment failures will be minor. Prospective study is needed to determine the validity of this conclusion.

## Data Availability

The datasets used and/or analyzed during the current study are available from the corresponding author upon reasonable request.
